# Association of histological features with laryngeal squamous cell carcinoma recurrences: a population-based study of 1502 patients in the Netherlands

**DOI:** 10.1186/s12885-022-09533-0

**Published:** 2022-04-22

**Authors:** Lilian N. Ruiter, Boukje A. C. van Dijk, Annette H. Bruggink, Patricia A. H. Doornaert, Marielle E. P. Philippens, Remco de Bree, Carla H. van Gils, Stefan M. Willems

**Affiliations:** 1 Department of Pathology, University Medical Center Utrecht, Utrecht University, Heidelberglaan 100, Utrecht, 3584 CX the Netherlands; 2 Department of Research and Development, Netherlands Comprehensive Cancer Organisation (IKNL), Utrecht, the Netherlands; 3 Department of Epidemiology, University Medical Center Groningen, University of Groningen, Groningen, the Netherlands; 4 Nationwide Network and Registry of Histo- and Cytopathology in the Netherlands (PALGA Foundation), De Bouw 123, Houten, 3991 SZ the Netherlands; 5 Department of Radiotherapy, University Medical Center Utrecht, Utrecht University, Heidelberglaan 100, Utrecht, 3584 CX the Netherlands; 6 Department of Head and Neck Surgical Oncology, University Medical Center Utrecht, Utrecht University, Heidelberglaan 100, Utrecht, 3584 CX the Netherlands; 7 Julius Center for Health Sciences and Primary Care, University Medical Center Utrecht, Utrecht University, Heidelberglaan 100, Utrecht, 3584 CX the Netherlands; 8 Present address: Department of Pathology, University Medical Center Groningen, University of Groningen, Hanzeplein 1, Groningen, 9713 GZ the Netherlands

**Keywords:** Laryngeal neoplasms, Squamous cell carcinoma, Recurrence, Histology, Pathology, Survival

## Abstract

**Background:**

Recurrences remain an important problem in laryngeal squamous cell carcinoma. Little has been described about histological characteristics of the primary laryngeal tumor that may be associated with recurrences. Identifying risk factors for recurrences might help in adapting treatment or follow-up. Using real-life population-based data, we aimed to identify histological features of the primary tumor associated with recurrences and overall survival.

**Material and methods:**

Demographic, clinical and treatment information on all first primary invasive laryngeal tumors diagnosed in 2010–2014 (*N* = 3705) were extracted from the population-based nationwide Netherlands cancer registry (NCR) and linked to PALGA, the nationwide Dutch pathology registry, to obtain data on histological factors and recurrences. For a random 1502 patients histological information i.e., keratinization, perineural invasion (PNI+), vascular invasion (VI+), growth pattern, degree of differentiation, extracapsular spread (ECS+), cartilage- and bone invasion and extralaryngeal extension, was manually extracted from narrative pathology reports and analyzed for locoregional recurrence and overall survival using cox regression analysis.

**Results:**

In total, 299 patients developed a locoregional recurrence and 555 patients died. Keratinization (HR = 0.96 (95%CI: 0.68–1.34) *p* = 0.79), two or three adverse characteristics (PNI+, VI+, non-cohesive growth) (HR = 1.38 (95% CI: 0.63–3.01) *p* = 0.42), and ECS+ (HR = 1.38 (95% CI: 0.48–4.02) *p* = 0.55) were not associated to recurrence. For death, also no significant association was found.

**Conclusion:**

In this population-based real-life dataset on laryngeal carcinoma in the Netherlands, histological factors were not associated with locoregional recurrences or overall survival, but future studies should investigate the role of these features in treatment decisions.

**Supplementary Information:**

The online version contains supplementary material available at 10.1186/s12885-022-09533-0.

## Introduction

In the last decades, survival rates of patients with laryngeal cancer were stable in The Netherlands with a 5-year survival of 70%, while survival improved for other head and neck cancers, such as oral cavity, oropharynx and hypopharynx tumors [1, 2]. The organ-preserving treatment regime of the last decades developed to improve the quality of life in patients with advanced disease might partially explain the stable survival rates of laryngeal cancers. It is suggested that this may be related to changes in patterns of management [3]. High recurrence rates could also contribute to this finding. According to literature, 22–31% [4, 5] of patients with primary laryngeal squamous cell carcinoma (LSCC) develop locoregional recurrences within 2 to 3 years after end of treatment [4, 6].

According to the national treatment guidelines, some tumor related characteristics are found associated with a higher chance of recurrences in head and neck cancer and adjuvant treatment is advised: positive resection margin (< 1 mm), close resection margin (1–5 mm), perineural invasion (PNI+), extralaryngeal extension, cartilage/bone invasion and extracapsular spread (ECS+) [7]. However, these guidelines are based on literature of head and neck tumors in general. Population-based studies describing associations between histological characteristics and recurrences in laryngeal cancer are lacking. If risk factors for recurrent disease can be identified, treatment can be more individualized and potentially (de-)escalated. Furthermore, follow-up visits can be more individualized based on these risk factors. The goal of this study is to identify histological features of the primary tumor associated with recurrences. In this same cohort, overall survival was also analyzed.

## Methods

### Study design

This population-based cohort study was based on Netherlands Cancer Registry (NCR) data. Patients diagnosed with laryngeal cancer between January 1, 2010 until December 31, 2014 were included and linked to pathology records from the national pathology archive (PALGA) via a third trusted party (ZorgTTP, Houten, The Netherlands) using pseudonyms of last name, date of birth and gender. PALGA (nationwide network and registry of histopathology and cytopathology in the Netherlands) contains all histopathology and cytology reports of the Netherlands [8]. The NCR (Netherlands Cancer Registry) records demographic variables, clinical variables and treatment of all Dutch cancer patients. This study does not fall under the scope of the Medical Research Involving Human Subjects Act (WMO). All data were handled according to GDPR.

### Inclusion / exclusion flowchart

**Fig. 1:**
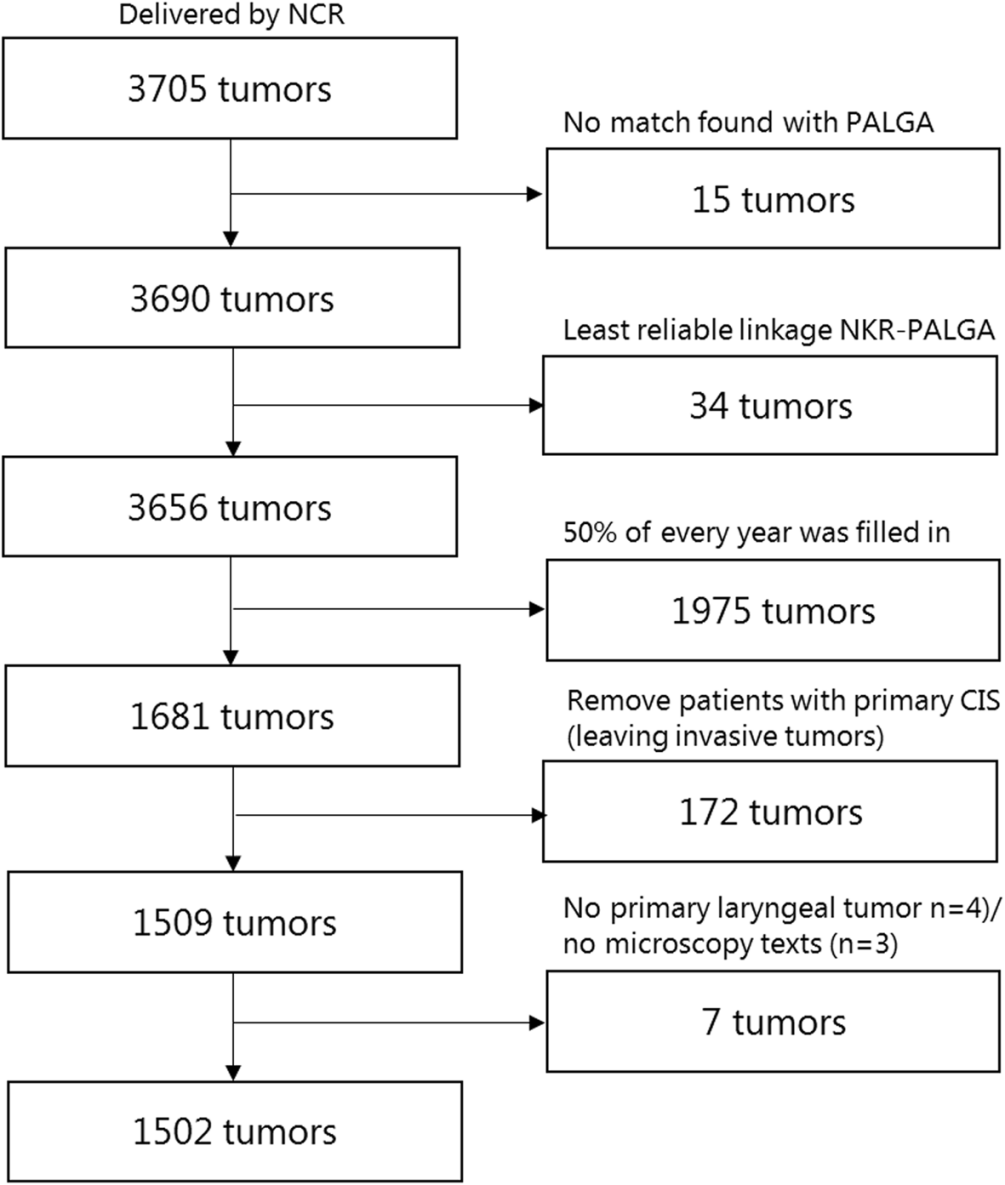
Flowchart of tumors included in the study. Tumors were excluded when: linking of the databases resulted in no matches or not very reliable matches. It was impossible to screen all records adequately, therefore, of every inclusion year (2010–2014) 50% of the pathology data were extracted of pathology reports. CIS patients were excluded. Only patients with invasive primary laryngeal squamous cell carcinoma with microscopy texts were included. Representativeness of the sample compared to the cohort was analyzed (Supplementary Table 1)

This population-based cohort consists of all Dutch patients with primary carcinoma in situ (CIS) of the larynx and LSCC without distant metastases at diagnosis between January 1, 2010 and December 31, 2014 in the Netherlands. In Fig. 1, a flowchart is included for the cleaning process of the research database. Three thousand seven hundred five tumors were delivered by the NCR and matched with PALGA. For 15 tumors no match was found with PALGA and for 34 tumors the match was unreliable, therefore these tumors were excluded, leaving 3656 tumors. Due to the labor intensity of extracting information out of narrative texts for every patient individually, it was impossible to screen all 3656 records adequately within a reasonable period of time. Therefore, we decided to evaluate 50% of the pathology records for every inclusion year (2010–2014). After removing patients with primary carcinoma in situ, leaving us 1509 invasive tumors. For 4 patients no pathology report for the primary laryngeal tumor was found and for 3 patients no microscopy text was available. Records for evaluation were randomly extracted; for the comparison of the patient and tumor characteristics for the total and selected population (Supplementary Table 1). Eventually, information on 1502 patients with invasive LSCC was included in this study.

### Determinants of interest

Pathology records were manually screened to record histological characteristics of the primary tumor and to identify recurrences. Histological factors of interest were perineural invasion (PNI+), vascular invasion (VI+), keratinization, growth pattern and degree of differentiation (well, moderately or poorly differentiated). In addition, extracapsular spread (ECS+) was investigated in a sub-analysis of patients with positive lymph nodes. For patients treated with total laryngectomy (TLE) another sub-analysis was performed for cartilage and bone invasion and extralaryngeal extension.

Since we were concerned about the correlation between variables of interest, we examined these possible relations, and decided that in case of relevant and significant correlation, we would build a new variable in which the number of adverse characteristics is used (one adverse characteristic - either VI+, PNI+ or non-cohesive growth pattern, two or three adverse characteristics or unknown) to be used in the multivariable analysis. A similar composite variable has been used for head and neck cancer previously [9]. This combined new variable was used for the multivariable analysis together with keratinization. Degree of differentiation was not added to the multivariable analysis as it was significantly associated with stage, which is included as a confounder. All histological factors of interest were scored based on pathology excerpts. An experienced head and neck pathologist (S.W.) was consulted in case of doubt.

### Outcome measures

The outcome measures were pathology-confirmed locoregional recurrences and death. Recurrences were marked as follows: the presence of LSCC or CIS locally or regionally within at least 2 years after treatment [10, 11]. CIS was included as a recurrence since CIS recurrences are (surgically) treated, which may affect the chance of developing a first invasive recurrence for these patients.

Time to locoregional recurrence was defined as the interval between first pathology report defining LSCC and the first pathology-confirmed local or regional recurrence. Patients were followed until locoregional recurrence, death or end-of-follow-up (20 October 2017) whichever occurred earlier. Time to death was the time interval between the first pathology excerpt confirming LSCC and death from any cause. Patients were censored when they were alive at the end of follow up.

### Confounders

All factors known to be associated with recurrence or death i.e. age, gender, sublocalisation and a combined variable for stage/treatment (Table 1) were included in the multivariable analysis. Since we were concerned of potential relevant and/or significant correlation between confounders of interest, we examined these possible relations. Stage and treatment were associated to each other and therefore combined into a new variable with clinically relevant patient groups referred to as “stage/treatment” (Table 1).

**Table 1:** Patient characteristics, locoregional recurrences and death of all patients with laryngeal squamous cell carcinoma (*n* = 1502) between 2010 and 2014 with at least 2 year follow-up

	Total		Recurrences			Death		
Characteristics	N	Column %	N	Row %	***P***-value	N	Row %	***P***-value
**Total**	1502	100	299	20		555	37	
**Sex**					‡			‡
M	1210	81	252	21	0.069	445	37	0.776
F	292	19	47	16		110	38	
**Age at diagnosis**								
Median (p25-p75)	66 (60–74)		64 (57–72)			70 (63–78)		
Mean (±SD)	67 ± 11		64 ± 11			70 ± 10		
**Localization**					†			†
Glottis	964	64	181	19	0.183	266	28	< 0.001
Supraglottis	509	34	110	22		265	52	
Subglottis	20	1	4	20		17	85	
Overlapping / unknown^a^	9	0.6	4	44		7	78	
**T clinical status**					†			~
T1	608	41	106	17	0.068	151	25	< 0.001
T2	392	26	90	23		144	37	
T3	322	21	75	23		167	52	
T4	148	10	23	16		85	57	
IS^b^	12	0.8	3	25		0	0	
TX^c^	20	1.3	2	10		8	40	
**N clinical status**					‡			‡
N0	1135	76	214	19	0.279	351	31	< 0.001
N1	94	6	22	23		51	54	
N2	168	11	43	26		115	68	
N3	9	0.6	2	22		6	67	
NX^c^	96	6	18	19		32	33	
**T pathological status**^d^					†			†
T1	237	46	41	17	0.020	51	22	< 0.001
T2	14	3	3	21		3	21	
T3	22	4	2	9		14	64	
T4	83	16	8	10		36	43	
TX^c^	155	30	40	26		42	27	
**N pathological status**^e^					†			‡
N0	65	13	7	11	0.037	19	41	< 0.001
N1	20	4	1	5		9	45	
N2	26	5	2	8		19	73	
NX^c^	400	78	84	21		99	25	
**Stage/treatment**					‡			~
T1/T2N0 Surg. (+RT/syst. therapy)	318	21	66	21	< 0.001	64	20	< 0.001
T1/T2N0 RT	512	34	84	16		141	28	
T1/T2N+ RT	71	5	25	35		42	59	
T3/T4N0/N+ Surg. (+RT/syst. therapy)	120	8	16	13		64	53	
T3/T4N0/N+ RT+/− syst. therapy)	318	21	79	25		152	48	
Other	163	11	29	18		92	56	

^a^ Overlapping or unknown: ICD-O-3: C32.8 or C32.9

^b^ IS: tumors initially clinically assessed as CIS (Carcinoma In Situ), but based on PA-tissue assessed as invasive carcinoma

^c^ TX/NX: T/N stage cannot be assessed

^d^ Only surgery patients (*n* = 511)

^e^ Only patients with PA-material of lymph nodes (*n* = 511)

† Fisher’s exact test

‡ Chi square test

~ Monte Carlo simulation based on 10^3^ sampled tables

### Statistical analysis

The association between different histological factors of interest and recurrence or death was investigated by a Chi square test or Fisher’s exact test. Categorical variables were represented as frequencies and percentages. Age was the only continuous variable and expressed as median with 25th/ 75th percentile. Chi square test was used for comparing recurrence rates for categorical variables. If conditions were not met, Fisher’s Exact test was used or Monte Carlo simulation was used (whichever was appropriate).

The proportional hazard assumption was assessed using log minus log plots and was met for all histological features of interest in relation to both outcomes. Univariable and multivariable analyses were performed by using Cox regression analysis for association of histological factors with time-to-recurrence or death. We reported hazard ratios (HRs) with their 95% confidence intervals (CIs) and *p*-values.

In the multivariable analysis, the number of adverse histological characteristics (VI+, PNI+ or non-cohesive growth pattern) and keratinization, were analyzed with correction for age, gender, sublocalisation and stage/treatment.

Subgroup analyses were performed for patients with positive lymph nodes: in this group separate multivariable analyses were performed to additionally assess ECS+ in relation to recurrences and death. Furthermore, a univariable subgroup analysis was performed for patients treated with total laryngectomy to additionally assess extralaryngeal growth and cartilage/bone invasion in relation to recurrences*.* However, a multivariable analysis could not be performed in this subgroup because of too few events.

IBM SPSS statistics version 25.0.0.2 was used for all statistical analyses, a *p*-value of < 0.05 was considered to be statistically significant.

## Results

In total, 299 (20%) patients developed a local (*n* = 215, 72%), regional (*n* = 69, 23%) or locoregional (*n* = 15, 5%) recurrence. Of the local recurrences, 20 (9%) were diagnosed as CIS and the rest as invasive carcinoma. Additionally, 6% of all patients developed pathological confirmed distant metastases.

Among patients with positive lymph nodes at diagnosis, 26% developed a recurrence (36% local, 58% regional and 6% locoregional recurrence) compared to 19% recurrences (80% local, 15% regional, 5% locoregional) among patients without positive lymph nodes at diagnosis.

### Clinical characteristics

The majority of patients diagnosed with primary laryngeal cancer was male; male patients also developed slightly more often a recurrence after treatment (*p* = 0.069), but no significant differences were found in mortality (*p* = 0.776). Within the follow-up period, a larger proportion of patients with supraglottic and subglottic cancer died compared to patients with glottic tumors (*p* < 0.001). For clinical and pathological TNM stage, clearly significant differences were observed in mortality (*p* < 0.001); however for recurrences, these differences were smaller. Only pathological T- and N-status were found to be significant associated with recurrences (*p* = 0.020) and (*p* = 0.037) respectively (Table 1).

### Histological characteristics

Growth pattern, VI+ and PNI+ were all significantly related to each other (p < 0.001). Correlations between growth pattern, VI+ and PNI+ varied between 0.51–0.70. Therefore, these PA-characteristics were combined into a new variable scoring 0 / 1 / ≥2 on adverse characteristics (non-cohesive growth / VI+ / PNI+). In addition, all histological characteristics (PNI+, VI+, growth pattern, keratinization, degree of differentiation, cartilage/bone invasion and extra laryngeal extension) were significantly associated to treatment (*p* ≤ 0.001).

PNI+, VI+, non-cohesive growth, the combined variable PNI+/VI+/Growth pattern, keratinization and degree of differentiation showed no significant differences in locoregional recurrence percentages. A significant higher proportion of patients with PNI+ (*p* = 0.001), VI+ (*p* = 0.036) or the combined variable PNI+/VI+/Growth pattern (*p* = 0.027) died (Table 2).

**Table 2:** Histological tumor characteristics, locoregional recurrences and death of all patients with laryngeal squamous cell carcinoma (*n* = 1502)

Histological features	Total		Recurrences			Death		
	N	Column %	N	Row %	***P***-value	N	Row %	***P***-value
**Perineural invasion**					†			†
No	1448	96	291	20	0.79	523	36	0.001
Yes	51	3	8	16		31	61	
Unclear / not evaluable	3	0.2	0	0		1	33	
**Vascular invasion**					†			†
No	1459	97	290	20	0.74	532	37	0.036
Yes	39	3	9	23		22	56	
Unclear / not evaluable	4	0.3	0	0		1	25	
**Growth pattern**					‡			‡
Cohesive growth	830	55	165	20	0.79	302	36	0.399
Non-cohesive growth	329	22	62	19		116	35	
Unclear / not evaluable	20	1	6	30		5	25	
Not determined	323	22	66	20		132	41	
**PNI+/VI+/Growth pattern**					‡			‡
No adverse characteristics	798	53	161	20	0.85	283	36	0.027
One adverse characteristics	318	21	58	18		111	35	
Two/three adverse characteristics	41	3	8	20		23	56	
One or more characteristics unknown	345	23	72	21		138	40	
**Keratinization**					‡			‡
No	212	14	44	21	0.88	81	38	0.664
Yes	867	58	174	20		312	36	
Unclear / not evaluable	26	2	5	19		11	42	
Not determined	397	26	76	19		151	38	
**Degree of differentiation**					‡			‡
Well differentiated (grade 1)	128	9	23	18	0.95	37	29	< 0.001
Moderately differentiated (grade 2)	595	40	118	20		220	37	
Poorly differentiated (grade 3)	214	14	43	20		110	51	
Unclear / not evaluable	4	0.3	0	0		2	50	
Not determined	561	37	115	21		186	33	

In a subset analysis of patients with positive lymph nodes, ECS+ was not related to different locoregional recurrence and death percentages (Supplementary Table 2).

In another subanalysis of patients treated with TLE, cartilage/bone invasion, extra laryngeal extension, PNI+, VI+, non-cohesive growth, the combined variable PNI+/VI+/Growth pattern, keratinization and degree of differentiation were not significantly different for locoregional recurrences. The proportion of deaths was significantly higher for patients with PNI+ (Supplementary Table 3).

### Survival analysis

The median follow-up time of all patients was 3.4 years and ranged from 7 days to 7 years. In total, 555 of the patients died. Two- and five-year cumulative survival rates were 79 and 60% respectively. Two-year LRC rate was 83%.

#### Locoregional recurrence

**Fig. 2:**
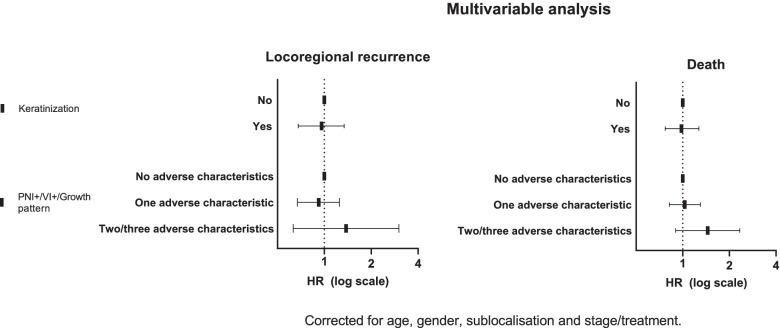
Multivariable analysis of histological characteristics of the primary tumor corrected for age, gender, sublocalisation and stage/treatment for locoregional recurrence and death *n* = 1502

In the multivariable analysis, keratinization showed no difference between non-keratinizing and keratinizing tumors for locoregional recurrences. Tumors containing two or three adverse PNI+/VI+/non-cohesive growth characteristics reveal a non-significantly higher HR (HR = 1.38 (0.63–3.01)) (Fig. 2).

A subanalysis was performed for patients with positive lymph nodes at diagnosis. Separate multivariable analyses revealed no significantly different HR for patients with ECS compared to patients with positive nodes without ECS (HR = 1.38 (0.48–4.02)) (Supplementary Figure 1).

A univariable subanalysis for TLE-patients revealed no difference in locoregional control for cartilage/bone invasion (HR = 1.74 (0.21–14.1) *p* = 0.61) and extralaryngeal extension (HR = 0.98 (0.24–3.93) *p* = 0.98) (Supplementary Figure 2). A multivariable analysis could not be performed in this subgroup because of too few events.

#### Overall survival

In the multivariable analysis, keratinizing tumors showed no difference in HR compared to non- keratinizing tumors (HR = 0.98 (0.77–1.27) *p* = 0.92). For two or three adverse PNI+/VI+/non-cohesive growth characteristics, the HR was not significantly increased for mortality (HR = 1.45 (0.90–2.33) *p* = 0.13) (Fig. 2).

In the subanalysis of patients with positive lymph nodes, separate multivariable analyses revealed no significant differences in HR for ECS+, keratinizing tumors and tumors containing two or three adverse PNI+/VI+/non-cohesive growth characteristics (Supplementary Figure 1).

For a univariable subanalysis of patients treated with TLE, only patients with PNI+ in their primary tumor had a significantly increased HR for mortality (HR = 2.10 (1.16–3.81) *p* = 0.014), whereas for cartilage/bone invasion and extralaryngeal extension no significantly increased HR was found (HR = 0.91 (0.45–1.84) *p* = 0.78) and (HR = 1.54 (0.84–2.82) *p* = 0.16) respectively (Supplementary Figure 2).

## Discussion

This population-based cohort study in the Netherlands was conducted to investigate whether specific histological features of primary laryngeal tumors are associated with recurrences and death. In total, 20% of the patients developed a locoregional recurrence and 37% of the patients died within a median follow-up period of 3.4 years.

Keratinization, two or three adverse characteristics (PNI+, VI+, non-cohesive growth) and ECS+ were not associated to locoregional recurrences and, in the multivariable analysis, not to death. In a subanalysis for patients treated with TLE, only PNI+ was significantly associated with death in a univariable analysis.

Only a few reports investigated the association of histological characteristics with locoregional recurrences in laryngeal carcinoma. For locoregional control, most studies found that histological characteristics were not significantly associated. ECS+, VI+, PNI+, infiltrative growth and non-keratinizing tumors were not predictive in uni-or multivariate analyses [12–16]. However, in a few articles ECS+ and non-keratinizing tumors were associated to locoregional control in multivariable analyses [14–16].

For overall survival, ECS+, PNI+ and non-keratinizing tumors are associated with death in univariable models [16, 17]. Some studies showed that ECS+ and PNI+ remained associated in a multivariable model, but in other studies, this effect disappeared for ECS+ and non-keratinizing tumors [13–16]. Other studies found that ECS+, PNI+, VI+ and infiltrative growth were in fact not significantly related to mortality in a univariable model. The contradiction and inconsistency of the results in literature may be explained by the method used for the multivariable analysis. Some studies did not always correct for confounders (often because of small numbers of events) [12–15]. Other studies added too many variables in the multivariable analysis for the number of events [16]. One study did not show which variables were included in the multivariable analysis [17]. In addition, the study populations differed from our population, mostly only T4 patients receiving a TLE were included [12–14, 17].

The presence or absence of pathological features such as perineural invasion and vascular invasion could indeed depend on the type of histological material (i.e. biopsy vs resection specimen), since biopsies vary by site and volume and are known to be difficult to reflect the entire tumor landscape [18]. The a priori probability of finding above mentioned pathological features in biopsy material were smaller compared to surgical specimen and this may explain the absence of an association. In our study, data available to the treating physicians in daily practice was included i.e. either only pathological features from biopsy material or from the surgical specimen were used in our main analysis. In our analysis, we did not find a relation between histological features of the primary tumor and recurrences. Additionally in the subgroup analyses for patients treated with a TLE, similar results were found (i.e. no relation was found between histological characteristics and recurrences).

Our data was nationwide and population-based. Although not all pathology records were screened to extract data, the included data were representative for the entire cohort (Supplementary Table 1). The setting of other studies was all single center [12–17] and not population-based. Despite the large number of patients included in our study, some histological characteristics were rarely observed, which resulted in small numbers in some categories. Given the rarity and small effect sizes we observed, we did not expect that expanding data extraction would change our interpretation of the relevance of these characteristics.

Even with the help of an experienced head and neck pathologist (S.W.), the narrative nature of the pathology reports made it sometimes difficult to extract the necessary information. Extracting histological information only from pathology reports is difficult since the context, as present in a multidisciplinary team meeting, is missing. Other information about the course of the patients would have been helpful in particular situations. In addition, for growth pattern, keratinization and degree of differentiation approximately a quarter was missing. This underlines the importance of synoptic reporting of laryngeal cancer in the Netherlands. Synoptic reporting is important to increase the completeness of data elements, like histological characteristics [19–21]. An increase in completeness of data is valuable for research, as more complete data allows to better investigate the role of each factor. In 2018, synoptic reporting was implemented for head and neck cancer in the Netherlands.

In the current study, only pathology-confirmed recurrences were included, so we possibly missed non-pathology confirmed recurrences. Within our own clinic of 526 laryngeal/hypopharyngeal cancer patients between 1995 and 2011 with a follow-up until 2017 we found that only 10 patients (2%) had a clinical recurrence without proven biopsy. Patients did not undergo a biopsy to confirm a recurrence, due to patient comorbidity, the inability to perform salvage surgery, irresectability of locoregional disease or distant metastasis, or patient refusal. With this observation, we expect that our findings would not change our outcome if non-pathology confirmed recurrences, that might be missed, would be included.

For this study, death was based on data of the population register of The Netherlands, where no information is available about the specific death cause. Disease specific survival describing death due to illness as an event could have given us some additional insight in the relation between locoregional recurrences and overall survival.

An advantage of this study was the real-life population-based data, containing a large and comprehensive group of patients with high generalizability. At the same time, we had to rely on data collected in the past as part of the clinical process, so data were not dedicatedly collected to facilitate research.

There is a possibility that an increased chance of developing a locoregional recurrence based on aggressive histological characteristics could be obscured because additional treatment was given. The national treatment guidelines used at the time of this study stated that there is an indication for postoperative radiotherapy in case of close resection margins (1–5 mm) or positive resection margins (< 1 mm), PNI+, extralaryngeal extension, cartilage invasion, subglottic extension and ECS+ with concomitant chemotherapy (if applicable) in case of positive surgical margins and ECS+ to reduce the chance of locoregional recurrences [7]. We also found that all histological characteristics were significantly associated with treatment. However, based on the historical data in this study, it is not clear how standardized or uniform these indications were applied in clinical practice. Therefore, it is unclear how histological factors might have contributed to treatment choice and to which extent treatment might have altered the risk of recurrence. The limited effect of histological characteristics associated with recurrences, may be due to adjuvant treatment indicated by these adverse histological predictors. However, treatment was included in the multivariable model as a confounder.

We investigated a heterogeneous group of patients who received different types of treatment. Although the histological features cannot be assessed in exact the same way for resection specimen as for biopsy material, we found that in the subgroup analysis of patients treated with TLE, which is a homogenously treated patient group with the same type of histological material, the results were similar to our overall findings. I.e., only PNI was significantly associated with death in univariable analysis.

We know, however, that other factors are likely to be relevant in relation to the occurrence of a recurrence. Information about the use of tobacco and alcohol status during follow-up would be interesting for prognosis. Unfortunately in our database, we do not have information about these factors, but this could be interesting to look at in the future.

In context, other relevant features for the future could be tumor microenvironment, genetic or epigenetic alterations, circulating tumor free DNA or tumor cells.

An example for the tumor microenvironment could be hypoxia; it is known to be associated with radio resistance in tumors and to affect treatment outcome [22, 23]. Perhaps other aspects of the tumor microenvironment such as (lymphatic) vasculature, type of inflammation (pro-carcinogenic / anti- carcinogenic) or type of immune cells might also play a role in outgrowth of minimal residual cancer [24–29].

An example of these genetic alterations is the presence of a TP53 mutation, since this alteration is frequently found in HNC [30]. Monitoring circulating tumor free DNA or circulating tumor cells seems a promising method to evaluate response during and after treatment [31–33].

It is important to find features that help to identify tumors with high risk of recurrence so treatment and follow-up can be individualized. We did not find an association between histological features and recurrences; more complete information and information on the role of histological features in treatment decision making could provide better insight.

## Supplementary Information

**Additional file 1: Supplementary Table 1.** Generalizability sample of pathology data extracted from pathology reports compared to initial cohort. A). Basic clinical characteristics of patients for each year of the sample used in this paper (*n* = 1502). These are the 3656 patients of which 44% of each year pathology data were extracted and CIS were removed. B). Basic clinical characteristics of the total dataset of patients for each year (*n* = 3380). These are the 3656 patients with reliable linkage between NKR-PALGA minus 276 CIS patients leaving 3380 patients in total. **Supplementary Table 2.** Histological characteristics, locoregional recurrences and death of patients treated with tumor-positive lymph nodes (*n* = 205). **Supplementary Table 3.** Histological characteristics, locoregional recurrences and death of T3/T4 N0/N+ patients treated with TLE or TLE with RT (*n* = 98). **Supplementary Figure 1.** Separate multivariable analysis of extracapsular spread, keratinization and PNI+/VI+/Growth pattern. Each separate histological characteristic was corrected for age, gender, sublocalisation and stage/treatment for locoregional recurrence and death of patients with positive lymph nodes (n = 205). **Supplementary Figure 2.** Univariable analysis of histological characteristics of the primary tumor for locoregional recurrence and death of T3/T4 N0/N+ patients treated with TLE or TLE with RT (n = 98). * *p* < 0.05 ** No events, excluded from analysis.

## Data Availability

The data that support the findings of this study are available from PALGA (Dutch Pathology Registry) and IKNL (Netherlands Comprehensive Cancer Organization) but restrictions apply to the availability of these data, which were used under license for the current study, and so are not publicly available. Data are however available from the authors upon reasonable request and with permission of PALGA and IKNL.

## Abbreviations

NCR: Netherlands cancer registry
PALGA: Nationwide Dutch pathology registry
PNI +: Perineural invasion
VI +: Vascular invasion
ECS +: Extracapsular spread
LSCC: Laryngeal squamous cell carcinoma
WMO: Medical Research Involving Human Subjects Act
CIS: Carcinoma in situ
TLE: Total laryngectomy
