# Supplementary material for: Association of histological features with laryngeal squamous cell carcinoma recurrences: a population-based study of 1502 patients in the Netherlands
Source: BMC Cancer. 2022 Apr 22;22:444. doi: 10.1186/s12885-022-09533-0 (PMC9034596; doi:10.1186/s12885-022-09533-0)
Supplement: Supplementary file 1 — Additional file 1: Supplementary Table 1. Generalizability sample of pathology data extracted from pathology reports compared to initial cohort. A). Basic clinical characteristics of patients for each year of the sample used in this paper (n = 1502). These are the 3656 patients of which 44% of each year pathology data were extracted and CIS were removed. B). Basic clinical characteristics of the total dataset of patients for each year (n = 3380). These are the 3656 patients with reliable linkage between NKR-PALGA minus 276 CIS patients leaving 3380 patients in total. Supplementary Table 2. Histological characteristics, locoregional recurrences and death of patients treated with tumor-positive lymph nodes (n = 205). Supplementary Table 3. Histological characteristics, locoregional recurrences and death of T3/T4 N0/N+ patients treated with TLE or TLE with RT (n = 98). Supplementary Figure 1. Separate multivariable analysis of extracapsular spread, keratinization and PNI+/VI+/Growth pattern. Each separate histological characteristic was corrected for age, gender, sublocalisation and stage/treatment for locoregional recurrence and death of patients with positive lymph nodes (n = 205). Supplementary Figure 2. Univariable analysis of histological characteristics of the primary tumor for locoregional recurrence and death of T3/T4 N0/N+ patients treated with TLE or TLE with RT (n = 98). * p < 0.05 ** No events, excluded from analysis. [file 12885_2022_9533_MOESM1_ESM.docx]

**Supplementary tables**

**Supplementary table 1. Generalizability sample of pathology data extracted from pathology reports compared to initial cohort.** A). Basic clinical characteristics of patients for each year of the sample used in this paper (n=1502). These are the 3656 patients of which 44% of each year pathology data were extracted and CIS were removed. B). Basic clinical characteristics of the total dataset of patients for each year (n=3380). These are the 3656 patients with reliable linkage between NKR-PALGA minus 276 CIS patients leaving 3380 patients in total.

|  | **A**. Basic clinical characteristics of patients for each year of the sample used in this paper (n=1502). | | | | | | | | | | | | | **B.** Basic clinical characteristics of the total number of patients for each year (n=3380). | | | | | | | | | | | |
| --- | --- | --- | --- | --- | --- | --- | --- | --- | --- | --- | --- | --- | --- | --- | --- | --- | --- | --- | --- | --- | --- | --- | --- | --- | --- |
|  | 2010 |  | 2011 |  | 2012 |  | 2013 |  | 2014 |  | Total |  | | 2010 |  | 2011 |  | 2012 |  | 2013 |  | 2014 |  | Total |  |
| Characteristics | N | % | N | % | N | % | N | % | N | % | N | % | | N | % | N | % | N | % | N | % | N | % | N | % |
| **Gender** |  |  |  |  |  |  |  |  |  |  |  |  | |  |  |  |  |  |  |  |  |  |  |  |  |
| M | 226 | **80** | 271 | **80** | 235 | **79** | 233 | **82** | 245 | **83** | 1210 | **81** | | 508 | **79** | 604 | **82** | 546 | **81** | 521 | **80** | 529 | **80** | 2708 | **80** |
| F | 58 | **20** | 66 | **20** | 65 | **22** | 52 | **18** | 51 | **17** | 292 | **19** | | 137 | **21** | 136 | **18** | 132 | **19** | 137 | **21** | 130 | **20** | 672 | **20** |
| **Localization** |  |  |  |  |  |  |  |  |  |  |  |  | |  |  |  |  |  |  |  |  |  |  |  |  |
| Glottis | 181 | **64** | 226 | **67** | 177 | **59** | 192 | **67** | 188 | **64** | 964 | **64** | | 411 | **64** | 480 | **65** | 417 | **62** | 421 | **64** | 413 | **63** | 2142 | **63** |
| Supraglottis | 98 | **35** | 103 | **31** | 118 | **39** | 92 | **32** | 98 | **33** | 509 | **34** | | 225 | **35** | 244 | **33** | 244 | **36** | 228 | **35** | 231 | **35** | 1172 | **35** |
| Subglottis | 3 | **1** | 6 | **2** | 3 | **1** | 1 | **0.4** | 7 | **2** | 20 | **1** | | 6 | **1** | 13 | **2** | 13 | **2** | 8 | **1** | 10 | **2** | 50 | **2** |
| Overlapping  or unknown^1^ | 2 | **0.7** | 2 | **0.6** | 2 | **0.7** | 0 | **0** | 3 | **1** | 9 | **0.6** | | 3 | **0.5** | 3 | **0.4** | 4 | **0.6** | 1 | **0.2** | 5 | **0.8** | 16 | **0.5** |
| **Stage** |  |  |  |  |  |  |  |  |  |  |  |  | |  |  |  |  |  |  |  |  |  |  |  |  |
| 0* | 1 | **0.4** | 0 | **0** | 1 | **0.3** | 0 | **0** | 0 | **0** | 2 | **0.1** | | 1 | **0.2** | 0 | **0** | 1 | **0.3** | 0 | **0** | 0 | **0** | 2 | **0.1** |
| 1 | 106 | **38** | 141 | **42** | 125 | **42** | 120 | **42** | 121 | **41** | 613 | **41** | | 252 | **39** | 305 | **41** | 271 | **40** | 264 | **40** | 246 | **37** | 1338 | **40** |
| 2 | 69 | **24** | 67 | **20** | 54 | **18** | 59 | **21** | 59 | **20** | 308 | **21** | | 157 | **24** | 153 | **21** | 136 | **20** | 137 | **21** | 134 | **20** | 717 | **21** |
| 3 | 51 | **18** | 66 | **20** | 54 | **18** | 49 | **17** | 57 | **19** | 277 | **18** | | 110 | **17** | 124 | **17** | 123 | **18** | 124 | **19** | 148 | **22** | 629 | **17** |
| 4 | 56 | **20** | 63 | **18** | 64 | **21** | 53 | **18** | 57 | **19** | 293 | **20** | | 122 | **19** | 157 | **21** | 142 | **21** | 127 | **19** | 127 | **19** | 675 | **20** |
| X | 1 | **0.4** | 0 | **0** | 2 | **0.7** | 4 | **1.4** | 2 | **0.7** | 9 | **0.6** | | 3 | **0.5** | 1 | **0.1** | 5 | **0.7** | 6 | **0.9** | 4 | **0.6** | 19 | **0.6** |
| **Radiotherapy** |  |  |  |  |  |  |  |  |  |  |  |  | |  |  |  |  |  |  |  |  |  |  |  |  |
| No | 80 | **28** | 106 | **32** | 86 | **29** | 72 | **25** | 81 | **27** | 425 | **28** | | 179 | **28** | 224 | **31** | 202 | **30** | 168 | **26** | 178 | **27** | 951 | **28** |
| Yes | 204 | **72** | 231 | **69** | 214 | **71** | 213 | **75** | 215 | **73** | 1077 | **72** | | 466 | **72** | 516 | **69** | 476 | **70** | 490 | **74** | 481 | **73** | 2429 | **72** |
| **Surgery** |  |  |  |  |  |  |  |  |  |  |  |  | |  |  |  |  |  |  |  |  |  |  |  |  |
| No | 192 | **68** | 215 | **64** | 191 | **64** | 194 | **68** | 200 | **68** | 992 | **66** | | 441 | **68** | 475 | **64** | 428 | **63** | 448 | **68** | 452 | **68** | 2244 | **66** |
| yes | 92 | **32** | 122 | **36** | 109 | **36** | 91 | **32** | 96 | **32** | 510 | **34** | | 204 | **32** | 265 | **36** | 250 | **37** | 210 | **32** | 207 | **31** | 1136 | **34** |
| **Systemic therapy** |  |  |  |  |  |  |  |  |  |  |  |  | |  |  |  |  |  |  |  |  |  |  |  |  |
| No | 258 | **91** | 314 | **93** | 274 | **91** | 266 | **94** | 268 | **91** | 1380 | **92** | 594 | | **92** | 673 | **91** | 613 | **90** | 595 | **91** | 582 | **88** | 3057 | **90** |
| Yes | 26 | **9** | 23 | **7** | 26 | **9** | 19 | **7** | 28 | **10** | 122 | **8** | 51 | | **8** | 67 | **9** | 65 | **10** | 63 | **10** | 77 | **12** | 323 | **10** |
| **Total** | 284 |  | 337 |  | 300 |  | 285 |  | 296 |  | 1502 |  | 645 | |  | 740 |  | 678 |  | 658 |  | 659 |  | 3380 |  |

* 2 patients were scored as CIS based on IKNL data but based on PALGA data these tumors were invasive. Based on morphological code we categorized these as invasive.

**Supplementary table 2:** Histological characteristics, locoregional recurrences and death of patients treated with tumor-positive lymph nodes (n=205).

| **Histological features** | **Total** | | **Recurrences** | | | **Death** | | |
| --- | --- | --- | --- | --- | --- | --- | --- | --- |
|  | **N** | **Column**  **%** | **N** | **Row**  **%** | **P-value** | **N** | **Row**  **%** | **P-value** |
| **Extracapsular spread** |  |  |  |  | † |  |  | † |
| No | 169 | 82 | 48 | 29 | 0.214 | 97 | 57 | 0.529 |
| Yes | 34 | 17 | 5 | 15 |  | 23 | 68 |  |
| Unclear / not evaluable | 2 | 1 | 0 | 0 |  | 1 | 50 |  |
| **Perineural invasion** |  |  |  |  | ‡ |  |  | ‡ |
| No | 185 | 90 | 52 | 28 | 0.029 | 106 | 57 | 0.126 |
| Yes | 20 | 10 | 1 | 5 |  | 15 | 75 |  |
| **Vascular invasion** |  |  |  |  | † |  |  | ‡ |
| No | 192 | 94 | 52 | 27 | 0.191 | 113 | 59 | 0.999 |
| Yes | 13 | 6 | 1 | 8 |  | 8 | 62 |  |
| **Type of growth** |  |  |  |  | ‡ |  |  | ‡ |
| Cohesive growth | 110 | 54 | 31 | 28 | 0.308 | 65 | 59 | 0.599 |
| Non-cohesive growth | 55 | 27 | 10 | 18 |  | 30 | 54 |  |
| Not determined | 40 | 20 | 12 | 30 |  | 26 | 65 |  |
| **PNI+/VI+/Growth pattern** |  |  |  |  | ‡ |  |  | ‡ |
| No adverse characteristics | 98 | 48 | 30 | 31 | 0.134 | 56 | 57 | 0.730 |
| One adverse characteristics | 52 | 25 | 10 | 19 |  | 29 | 56 |  |
| Two/three adverse characteristics | 15 | 7 | 1 | 7 |  | 10 | 67 |  |
| One or more characteristics unknown | 40 | 20 | 12 | 30 |  | 26 | 65 |  |
| **Keratinization** |  |  |  |  | ‡ |  |  | ‡ |
| No | 40 | 20 | 11 | 28 | 0.864 | 26 | 65 | 0.243 |
| Yes | 105 | 51 | 28 | 27 |  | 56 | 53 |  |
| Unclear / not evaluable^1^ | 5 | 2 | 0 | 0 |  | 4 | 80 |  |
| Not determined^1^ | 55 | 27 | 14 | 26 |  | 35 | 64 |  |
| **Degree of differentiation** |  |  |  |  | ‡ |  |  | ‡ |
| Well differentiated  (grade 1) | 10 | 5 | 1 | 10 | 0.143 | 5 | 50 | 0.132 |
| Moderately differentiated  (grade 2) | 72 | 35 | 23 | 32 |  | 38 | 53 |  |
| Poorly differentiated  (grade 3) | 54 | 26 | 9 | 17 |  | 39 | 72 |  |
| Not determined | 69 | 34 | 20 | 29 |  | 39 | 57 |  |

^1^ For Chi square or Fisher’s exact test the group unclear/not evaluable and not determined were combined into one group.

**Supplementary table 3:** Histological characteristics, locoregional recurrences and death of T3/T4 N0/N+ patients treated with TLE or TLE with RT (n=98).

| **Histological features** | **Total** | | **Recurrences** | | | **Death** | | |
| --- | --- | --- | --- | --- | --- | --- | --- | --- |
|  | **N** | **Column**  **%** | **N** | **Row**  **%** | **P-value** | **N** | **Row**  **%** | **P-value** |
| **Cartilage/bone invasion** |  |  |  |  | † |  |  | ‡ |
| No | 20 | 20 | 1 | 5 | 0.999 | 10 | 50 | 0.681 |
| Yes | 78 | 80 | 7 | 9 |  | 35 | 45 |  |
| **Extra laryngeal extension** |  |  |  |  | † |  |  | † |
| No | 45 | 46 | 4 | 9 | 0.999 | 19 | 42 | 0.606 |
| Yes | 52 | 53 | 4 | 8 |  | 26 | 50 |  |
| Not determined | 1 | 1 | 0 | 0 |  | 0 | 0 |  |
| **Perineural invasion** |  |  |  |  | † |  |  | ‡ |
| No | 64 | 65 | 3 | 5 | 0.121 | 24 | 38 | 0.022 |
| Yes | 34 | 35 | 5 | 15 |  | 21 | 62 |  |
| **Vascular invasion** |  |  |  |  | † |  |  | † |
| No | 78 | 80 | 6 | 8 | 0.681 | 36 | 46 | 0.999 |
| Yes | 19 | 20 | 2 | 11 |  | 9 | 48 |  |
| Unclear / not evaluable | 1 | 1 | 0 | 0 |  | 0 | 0 |  |
| **Type of growth** |  |  |  |  | † |  |  | † |
| Cohesive growth | 35 | 36 | 2 | 6 | 0.549 | 17 | 49 | 0.385 |
| Non-cohesive growth | 57 | 58 | 5 | 9 |  | 27 | 47 |  |
| Not determined | 6 | 6 | 1 | 17 |  | 1 | 17 |  |
| **PNI+/VI+/Growth pattern** |  |  |  |  | † |  |  | † |
| No adverse characteristics | 19 | 19 | 1 | 5 | 0.284 | 8 | 42 | 0.195 |
| One adverse characteristics | 45 | 46 | 2 | 4 |  | 20 | 44 |  |
| Two/three adverse characteristics | 27 | 28 | 4 | 15 |  | 16 | 59 |  |
| One or more characteristics unknown | 7 | 7 | 1 | 14 |  | 1 | 14 |  |
| **Keratinization** |  |  |  |  | † |  |  | ‡ |
| No | 12 | 12 | 2 | 17 | 0.262 | 6 | 50 | 0.853 |
| Yes | 66 | 67 | 4 | 6 |  | 29 | 44 |  |
| Unclear / not evaluable^1^ | 1 | 1 | 0 | 0 |  | 1 | 100 |  |
| Not determined^1^ | 19 | 19 | 2 | 10 |  | 9 | 47 |  |
| **Degree of differentiation** |  |  |  |  | † |  |  | † |
| Well differentiated  (grade 1) | 5 | 5 | 0 | 0 | 0.257 | 2 | 40 | 0.266 |
| Moderately differentiated  (grade 2) | 46 | 47 | 2 | 4 |  | 17 | 37 |  |
| Poorly differentiated  (grade 3) | 32 | 33 | 4 | 13 |  | 19 | 60 |  |
| Not determined | 15 | 15 | 2 | 13 |  | 7 | 47 |  |

^1^ For Chi square or Fisher’s exact test the group unclear/not evaluable and not determined were combined into one group.

**Supplementary figures**

**Supplementary Figure 1.**

**Supplementary Figure 2.**
